# Fruit Pouch Consumption Does Not Associate with Early Manifestations of Allergic Disease

**DOI:** 10.3390/nu15204318

**Published:** 2023-10-10

**Authors:** Emmy Fredriksson, Stina Bodén, Magnus Domellöf, Christina E. West

**Affiliations:** Department of Clinical Sciences, Pediatrics, Umeå University, SE 90185 Umeå, Sweden; emmy.fredriksson@gmail.com (E.F.); stina.boden@umu.se (S.B.); magnus.domellof@umu.se (M.D.)

**Keywords:** allergic disease, asthma, eczema, epithelial barrier hypothesis, food allergy, fruit pouches, NorthPop, pediatrics, sensitization, wheeze

## Abstract

Consumption of acidic fruit pouches in infancy may damage the epithelial barrier in the gastrointestinal tract and is suggested to increase allergy risk. We aimed to explore if a high fruit pouch consumption is associated with a higher incidence of early allergic manifestations. We included 2959 parent–child dyads from the Swedish prospective, population-based NorthPop birth cohort study with parentally reported data on frequency of fruit pouch consumption at 9 months of age, as well as parentally reported eczema, wheeze, physician-diagnosed asthma, and food allergy in the first 18 months of life. Immunoglobulin E levels (IgE) in serum (*n* = 1792), as response to a food mix and an inhalant mix, were determined at age 18 months. Compared with no consumption, daily consumption of one or more pouches at 9 months of age was associated with inhalant sensitization (odds ratio (OR) 2.27, 95% confidence interval (CI) 1.06–4.87, *n* = 1792) but did not remain significant in the multivariable adjusted model (_a_OR 2.08, 95% CI 0.95–4.53, *n* = 1679). There were no associations between fruit pouch consumption and allergic manifestations at this young age. This study suggests that fruit pouch consumption is not associated with allergic phenotypes or IgE sensitization in early childhood.

## 1. Introduction

The prevalence of allergic diseases is increasing in low-, middle- and high-income countries [1]. Although studies reported that the increase reached a plateau in some parts of the world [2], allergic sensitization and allergic asthma are still on the rise in Sweden [3,4], where every third adolescent is affected by one or more allergic diseases [5]. This has negative consequences for the individual and their family, and the rise in prevalence has also increased the burden on healthcare systems and economies worldwide [1]. Thus, it is of major importance to study the causes behind this negative trend to develop better prevention and treatment strategies. The recently proposed epithelial barrier hypothesis states that damage done to the epithelial barrier can affect the development of allergic and autoimmune diseases not only locally but also systemically [6]. By damaging the epithelial barrier, the opportunity arises for microbial dysbiosis and translocation of bacteria, which in turn trigger an immune response that can lead to the development of chronic autoimmune, metabolic, and allergic diseases [6].

Adaptation to a Western standard of living, which includes extensive changes in dietary habits over the last decades, was implicated in the “allergy epidemic” [6]. The industry’s production of convenient food and food packages for infants as well as changes in dietary patterns contribute to the increased intake of fruit pouches, which are defined as pureed fruit packaged in a squeeze pouch [7]. Although food pouches have been on the market for over 20 years, the intake of these products increased over the last decade, to the point where in 2016, approximately one-third of American infants between the ages of 6–12 months consumed fruit pouches regularly [7].

It was recently proposed that the low pH value (<4) of commercial baby foods, particularly those with fruit or citric acid added, increases the risk of developing oesophageal diseases [8]. Fruit pouches have a low pH, and at the same time, these products do not stimulate chewing and do not promote the production of saliva, which is an important buffer when ingesting acidic foods [8]. Consequently, intake of acidic fruit pouches in the absence of the protective effect of saliva induced by chewing may damage the epithelial barrier. Hence, a relevant question to investigate is if the consumption of acidic food in infancy increases the risk of developing allergic diseases, as was suggested [8].

We hypothesized that intake of fruit pouches in infancy is associated with the development of allergic diseases in early life. The objective was to assess the associations between fruit pouch consumption and early allergic manifestations until 18 months of age in a large population-based birth cohort study.

## 2. Materials and Methods

### 2.1. Data Collection

This cohort study is based on data collected from the NorthPop birth cohort study, an ongoing prospective population-based study aiming to recruit 10,000 families in Västerbotten county, Sweden. Currently, there are over 8000 participating families [9].

During the appointment for the routine ultrasound examination at gestational weeks 17 to 20, the pregnant woman and her partner received oral and written information about this study and were invited to participate. To be eligible for participation in the NorthPop birth cohort study, the pregnant woman must have a viable pregnancy between gestational weeks 14 and 24 and be at least 18 years old. Additional inclusion criteria are the intention to give birth as well as stay in Västerbotten county during the forthcoming years. The pregnant woman must also be able to read and understand Swedish, since this is the only language supported by the NorthPop birth cohort study.

Data in this study were primarily collected through web-based questionnaires. Before the delivery of the child, the pregnant woman and her partner responded to questionnaires at the time of enrolment and at gestational weeks 26 and 35, respectively. The questions were primarily focused on socioeconomics, lifestyle, and medical history. After the child was born, the parents answered questionnaires when their child was 4, 9, and 18 months old, respectively. Information on infant feeding, specifically if the infant was exclusively breastfed, exclusively formula-fed, or mixed-fed, was collected from the 4-month questionnaires. At 9 months of age, we collected information on how frequently the infant consumed industrially produced fruit pouches. The incidence of allergic manifestations was assessed at 18 months of age. At that age, data were also collected from venous blood samples to determine if the child had developed immunoglobulin E (IgE) sensitization to inhalant and food allergens.

### 2.2. Study Population

**Figure 1:**
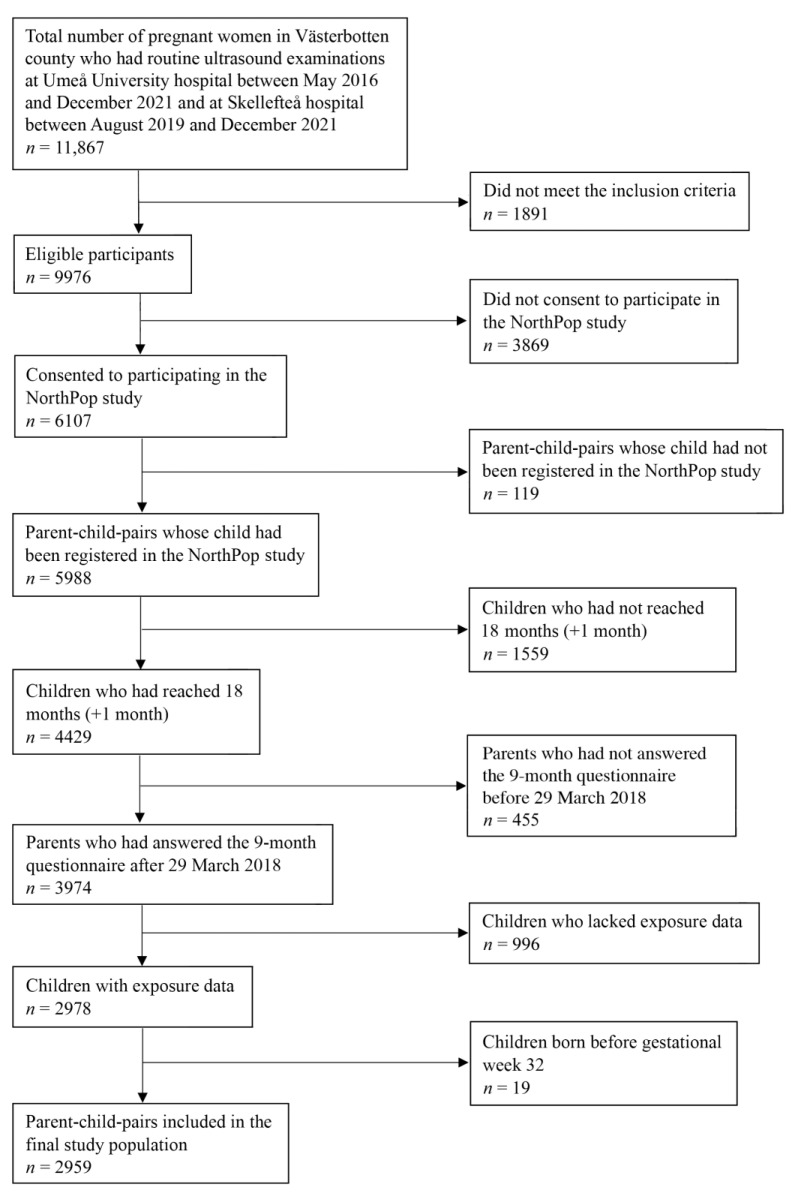
Flowchart of the study population.

Between May 2016 and December 2021, 11,867 pregnant women had their routine ultrasound examinations within the NorthPop study catchment area, i.e., Västerbotten county. A flowchart for inclusion and exclusion of study participants is depicted in Figure 1. This study is reported in accordance with Strengthening the Reporting of Observational Studies in Epidemiology and nutrition (STROBE-Nut) guidelines for observational studies.

### 2.3. Main Exposures

Information concerning the infant’s dietary patterns, including the frequency of fruit pouch consumption, was collected from a food frequency questionnaire (FFQ) at 9 months of age. The FFQ questions analyzed in this study can be found in Appendix A. Fruit pouches were defined as pureed fruit packaged in a squeeze pouch. The frequency of fruit pouch consumption was divided into six groups—never, 1–3 times per month, 1–3 times per week, 4–6 times per week, 1–3 times per day, and 4–6 times per day. Because of few respondents in the group consuming 4–6 pouches per day, we merged it with the group consuming 1–3 pouches per day.

### 2.4. Primary Outcomes

Primary outcomes were allergic manifestations in the child at 18 months of age, which in this study were defined as parentally reported eczema, wheeze, physician-diagnosed food allergy, physician-diagnosed asthma, and IgE sensitization in blood drawn at the study visit at 18 months of age. Eczema severity was further analyzed by using patient-oriented eczema measure scores (POEM). The POEM scores were dichotomized into two groups based on five clinically used severity bands: group 1, score 0–7 (clear to mild eczema), and group 2, score 8–28 (moderate to very severe eczema) [10]. 

As previously described [11], venous blood samples were collected in Vacutainer tubes at 18 months for determination of allergen-specific levels of IgE sensitization to inhalant (Phadiatop) and food allergens (food mix fx5: egg white, wheat, cow’s milk, codfish, peanut, and soybean). Determination was completed using ImmunoCAP, Thermo Fisher Scientific/Phadia, Uppsala, Sweden, according to the manufacturer’s instructions. A test was considered positive if IgE levels ≥ 0.35 PAU (Phadiatop) and ≥0.35 kUA/L (fx5).

### 2.5. Covariates

Data regarding the infant’s sex, birth weight and length, delivery method, and gestational week were collected from the Swedish pregnancy register [12]. Additional covariates, such as the infant’s diet at 4 months and the presence of siblings and pets in the first 9 months of life, were collected through self-reported NorthPop questionnaires at 4 and 9 months of age. Parental covariates considered in this study were age, education level, diet, smoking, asthma, and allergies, including hay fever, fur allergy, eczema, and food allergy. These covariates were also obtained from self-reported NorthPop questionnaires between pregnancy week 18 and 35. Education level was defined as either having an academic education or not. Additionally, the mother’s body mass index (BMI) when the pregnancy was registered at the first maternal health care appointment was collected from the Swedish pregnancy register [12]. 

### 2.6. Statistical Analysis

All statistical analyses were performed in SPSS Statistics version 28.0.1.1, and *p*-values < 0.05 were considered statistically significant. A chi-2 test was used to determine the association between children with a positive food mix test and children with food allergy diagnosis at 18 months. To assess possible associations between consumption frequency of fruit pouches and allergic manifestations, we first ran univariable logistic regression presenting odds ratios (OR) and 95% confidence interval (CI) per fruit pouch consumption level in four categories (1–3 per month, 1–3 per week, 4–6 per week, and 1 or more per day) compared to the reference group that reported no fruit pouch consumption. To determine possible confounders a priori, we used the tool DAGitty, version 3.0 [13] (Appendix B). Three potential confounders related to both the exposure and the outcomes were identified, i.e., parental education level, having siblings, and the infant’s diet (breastfeeding, formula feeding, or mixed feeding) at 4 months of age and were thus included in a multivariable logistic regression model.

## 3. Results

### 3.1. Baseline Characteristics

We included 2959 parent–child pairs, and their baseline characteristics are displayed in Table 1. We present characteristics from mother, child, and partner, except for the 37 pairs who did not have a partner included in this study. The mean (SD) age of the mothers was 31.0 (4.3) years, while the mean age for the partners was 32.9 (6.6) years. In all, 68.6% of the mothers and 44.5% of the partners had a university degree.

### 3.2. Fruit Pouch Consumption at 9 Months of Age

**Figure 2:**
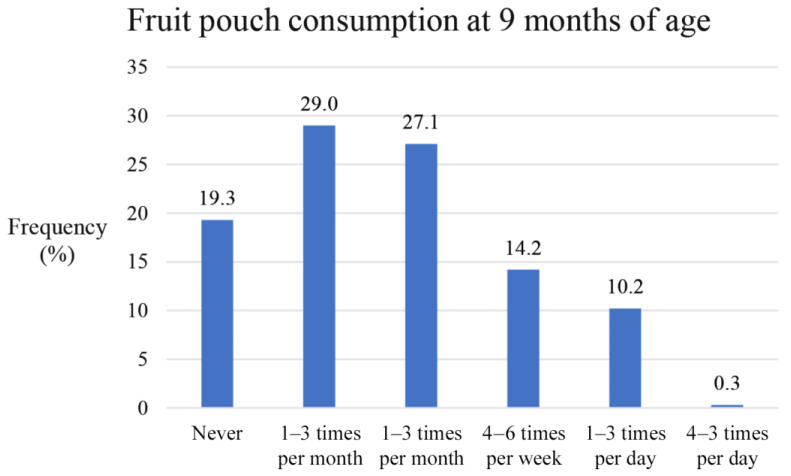
Fruit pouch consumption shown as percentages at nine months of age in 2959 children, grouped by frequency of consumption.

The 9-month FFQ showed that 19.3% of the 2959 children did not consume any fruit pouches, while 80.7% consumed fruit pouches ranging from rarely (one to three times per month) to very frequently (four to six times per day) (Figure 2). 

### 3.3. Prevalence of Allergic Diseases at 18 Months of Age

The number of responses varied between questions regarding outcome. Eczema, asthma, and wheeze had 2648, 2649, and 2650 responses, respectively. For food allergies, there were a total of 2252 responses, while adverse symptoms provoked by food had two additional responses, 2254 in total. Meanwhile, 1792 children provided samples that were tested for IgE sensitization.

**Figure 3:**
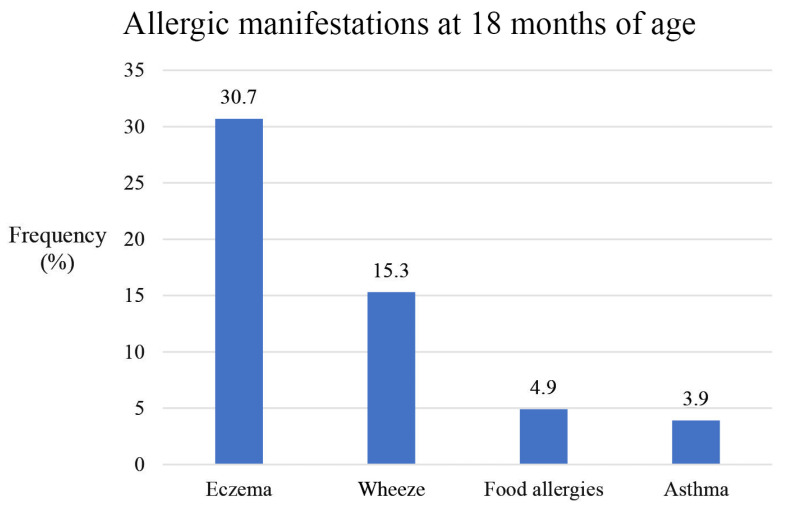
Allergic manifestations shown in valid percentages at 18 months of age. Eczema, asthma, and wheeze had 2648, 2649, and 2650 responses, respectively. For food allergies, there were a total of 2252 responses.

Eczema had the highest prevalence, followed by wheeze, physician-diagnosed food allergy, and, finally, physician-diagnosed asthma (Figure 3). In total, 813 (30.7%) children were reported to have eczema, of whom 88 children (3.4% of the total study population) were classified to have moderate to severe eczema according to POEM scores (8–28 points out of 28). Any adverse symptoms provoked by food was reported in 22.9% (*n* = 516) of children compared to 4.9% (*n* = 111) who had been diagnosed with a food allergy by a physician. Overall, 17.1% (*n* = 306) of the children who provided samples were sensitized to food allergens, while 4.6% (*n* = 83) were sensitized to inhalant allergens at 18 months of age. Among the food-allergic children, 40 out of 83 (48.2%) had a positive food mix fx5 test versus 223 out of 1500 (14.9%) among those without a food allergy diagnosis (*p* < 0.001). The corresponding number for inhalants were 11 out of 83 (13.3%) and 60 out of 1500 (4.0%) (*p* < 0.001).

### 3.4. Associations between Fruit Pouch Consumption and Early Allergic Manifestations

**Figure 4:**
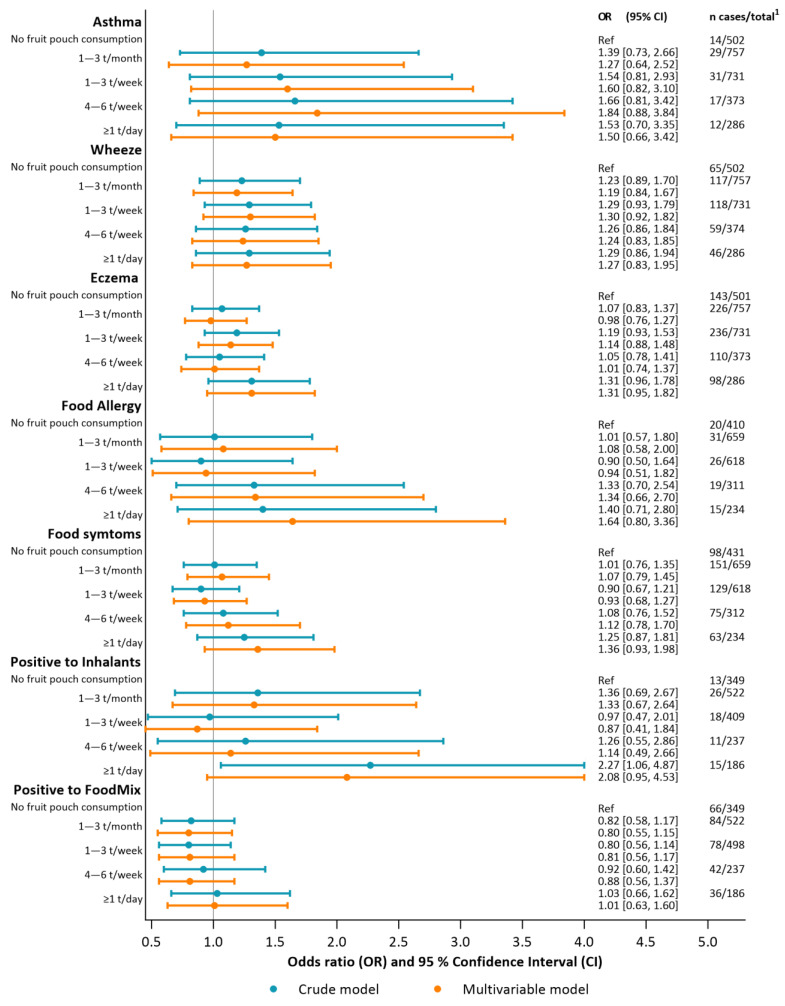
Associations between fruit pouch consumption and allergic manifestations presented in a crude (blue) and a multivariable (orange) model. For each outcome, four categories of consumption are compared to the reference group that reported no fruit pouch consumption. ^1^ refers to the total number of participants in the crude model. *n* = number, t = times.

A crude logistic regression of fruit pouch consumption at 9 months of age did not show any association with the prevalence of wheeze, asthma, eczema, or food allergy at 18 months of age, regardless of the amount of fruit pouches consumed. An analysis of adverse symptoms related to food and drinks and food sensitization at 18 months also showed nonsignificant results. However, when analysing IgE sensitization to inhalant allergens in a crude logistic regression, a significant association was found for the group consuming one or more fruit pouches per day (OR 2.27, 95% CI 1.06–4.87, *n* = 1792), see Figure 4.

When adjusting for the three potential confounding factors, parental education level, having siblings, and the infant’s diet at 4 months of age (breastfeeding or formula feeding), the odds of developing IgE sensitization to inhalant allergens were still increased for infants with higher fruit pouch consumption, but the association was no longer statistically significant (OR 2.08, 95% CI 0.95–4.53, *n* = 1679) (Figure 4). For the other outcomes, there were no associations with the intake of fruit pouches in the multivariable models.

## 4. Discussion

To the best of our knowledge, this is the first study investigating the possible associations between fruit pouch consumption and allergic manifestations in young children. In the unadjusted models, we found that high fruit pouch consumption, defined as daily consumption of one or more fruit pouches, at age 9 months was significantly associated with an increased risk of IgE sensitization to inhalants at 18 months of age, but the association did not remain statistically significant after multivariable adjustment. In contrast to our hypothesis, there were no significant associations between high fruit pouch consumption and increased risk of early manifestations of allergic diseases, despite observing a higher consumption of fruit pouches than previous studies in 9-month-olds [7]. Hence, our results do not support the hypothesis that high fruit pouch consumption affects allergy risk, at least not as early as at 18 months of age.

According to the epithelial barrier hypothesis, epithelial damage creates a leaky epithelium, where the dead epithelial cells, other inflammatory cells, and cytokines are flushed away to reduce the local epithelial burden [14]. This allows all microbial content to pass deeper into the tissue, where it triggers a skewed immune response [14]. Damage to the epithelium also creates a space for opportunistic bacteria to colonize the area, which further exacerbates the problem by influencing the composition and diversity of the microbiome, which has been linked to the development of inflammatory and allergic diseases [6,15,16,17,18]. 

It was theorized that the acidity of fruit pouches may damage the epithelial barrier in the gastrointestinal tract and trigger an immune response [8]. Notably, the single study that measured the pH of the most commonly consumed baby pouches in Australia found that all pouches containing only fruit were markedly acidic (pH < 4) [8]. Other studies showed that a pH < 4 in the esophagus may cause epithelial damage [8]. Worth noting, however, is that no similar studies have been performed on the pH levels of fruit pouches sold in Sweden. Although the positive association between fruit pouch consumption and IgE sensitization to inhalants did not remain in the multivariable model, future studies at an older age when inhalant sensitization is more common would be valuable. In regions of high exposure to grass pollen, allergic individuals are commonly sensitized to profilin, and of these, some have reactions to profilin in foods, e.g., fruits [19]. In adults, there is some evidence that disruption of epithelial integrity of the gastrointestinal mucosa allows profilin to enter the mucosa and induce local inflammation [20]. The lack of association between high fruit pouch consumption and allergic diseases in our study is likely also influenced by protective factors, including dietary factors such as breastfeeding and additional nutritive factors in the infant diet [21,22]. Dietary patterns rich in fiber from fruit, which positively affects the alpha diversity of the developing microbiota [23,24], may also be protective [25].

In this study, we hypothesized that a low pH might be associated with the development of allergic disease and asthma. On the other hand, there is emerging evidence that regular use of proton-pump inhibitors (PPI) in children resulting in a higher pH in the gastrointestinal tract is in fact associated with an increased risk of asthma [26,27]. Studies also showed association between the use of acid suppressive medications in children and an increased risk of other allergic diseases, such as food allergy, anaphylaxis, and allergic rhinitis [28,29]. Hence, there are possible adverse effects to be considered regarding factors that decrease the acidity in the gut.

The mode of feeding of fruit pouches, for which information is not obtained in the NorthPop FFQs, might also have an impact on the results of this study. Studies investigating the effects of sipping the purée directly from the pouch versus squeezing the contents onto a spoon would be of value, and one such study is currently underway in New Zealand [30]. Furthermore, comparing how a lack of chewing and a simultaneously increased acidity affects the total production of saliva is also of interest. While some studies on the nutritional content of fruit pouches were conducted in the United States, they were primarily focused on the amount of sugar and therefore lack relevant information on the addition of acidic ingredients and pH levels in the pouches [31,32]. Also, there has been concern that the high sugar content of fruit pouches may predispose infants to excessive weight gain [33,34]. Reassuringly, a previous study from the NorthPop birth cohort study reported that fruit pouch consumption was not associated with excess weight gain in the first 18 months of life [35].

In our population, 51.5% of the 9-month-olds consumed one or more fruit pouches per week. This is a higher average than for the 6- to 12-month-olds presented in a previous American study where a third of the infants consumed one or more fruit pouches per week [7]. 

Strengths of this study include the large study sample that was collected from a well-designed, prospective, population-based study, providing a diverse range of data that are less susceptible to confounding compared to retrospective studies. The data collected on many potential confounders facilitated the construction of a directed acyclic graph. However, a possible limitation is that the data were self-reported, which may increase the risk for recall bias as well as under- and overreporting of food consumption. Another limitation was the dropouts from recruitment until the age of 18 months of the child. Participation in the IgE serology study was also noted to be higher in mothers with high educational level [11], making the study population less representative. 

## 5. Conclusions

In this large population-based birth cohort study, no associations were found between a high fruit pouch consumption and early allergic manifestations that remained statistically significant in the multivariable models. Moreover, a previous NorthPop study reported no association between high fruit pouch consumption and excessive weight gain [35]. Consequently, the combined findings from these two studies imply that fruit pouch consumption is safe for young children in terms of growth and allergy risk. However, since inhalant sensitization in early life is a risk factor for allergic airway diseases [36], the ongoing follow-up of the NorthPop birth cohort study will allow future studies to determine the impact of fruit pouch consumption and dietary patterns on the risk of developing allergic diseases until school age.

## Figures and Tables

**Table 1:** Baseline characteristics for child, mother, and partner.

**Child Characteristics**				
	**Child, *n* = 2959**		**Missing Data, Child**	
	**N (%) ^1^/Mean (SD) ^2^**		**N (%) ^1^**	
**Girls**	1441 (48.7) ^1^		0 (0) ^1^	
**Birth weight**	3536.0 (522.9) ^2^		0 (0) ^1^	
**Birth length**	50.11 (2.5) ^2^		0 (0) ^1^	
**Delivery method** Vaginal Caesarean section Obstetrical vacuum extraction	2303 (77.8) ^1^ 515 (17.4) ^1^ 135 (4.6) ^1^		0 (0) ^1^	
**Gestational week**	39.33 (1.6) ^2^		0 (0) ^1^	
**Siblings (yes)**	1165 (39.9) ^1^		150 (5.1) ^1^	
**Feeding at 4 months** Breastfeeding Breastfeeding and formula Formula	1883 (63.6) ^1^ 450 (15.2) ^1^ 489 (16.5) ^1^		137 (4.6) ^1^	
**Pets in first 9 months of life (yes)**	1576 (53.2) ^1^		145 (4.9) ^1^	
**Blood sample taken at 18 months**	1792 (60.6) ^1^		1167 (39.4) ^1^	
**Parental characteristics**				
	**Mother, *n* = 2959**	**Missing data, mother**	**Partner, *n* = 2922**	**Missing data, partner**
	**N (%) ^1^/Mean (SD) ^2^**	**N (%) ^1^**	**N (%)^1^/Mean (SD) ^2^**	**N (%) ^1^**
**Age**	31.0 (4.3) ^2^	0 (0) ^1^	32.9 (6.6) ^2^	0 (0) ^1^
**Education** <9 years High school University	74 (2.5) ^1^ 802 (27.1) ^1^ 2029 (68.6) ^1^	54 (1.8) ^1^	64 (2.2) ^1^ 1144 (38.7) ^1^ 1316 (44.5) ^1^	435 (14.7) ^1^
**Restricted diet ^3^**	407 (13.7) ^1^	94 (3.2) ^1^	182 (6.1) ^1^	532 (18.0) ^1^
**BMI**	24.9 (4.6) ^2^	72 (2.4) ^1^	-	-
**Smoking** No Last month before pregnancy During pregnancy	2783 (94.1) ^1^ 70 (2.4) ^1^ 38 (1.3) ^1^	68 (2.3) ^1^	-	-
**Asthma (yes)**	543 (18.4) ^1^	61 (2.1) ^1^	426 (14.4) ^1^	467 (15.8) ^1^
**Hay fever (yes)**	788 (26.6) ^1^	61 (2.1) ^1^	732 (24.7) ^1^	467 (15.8) ^1^
**Fur allergy (yes)**	598 (20.2) ^1^	61 (2.1) ^1^	645 (21.8) ^1^	467 (15.8) ^1^
**Food allergy (yes)**	490 (16.6) ^1^	61 (2.1) ^1^	253 (8.6) ^1^	467 (15.8) ^1^
**Eczema (yes)**	477 (16.1) ^1^	61 (2.1) ^1^	180 (6.1) ^1^	467 (15.8) ^1^

^1^ Number of samples (percentage of total population)*,* ^2^ mean within the total population shown with the standard deviation (SD)*,* ^3^ restricted diet categories include pescetarian, nonpork, nonfish, lacto-ovo-vegetarian, vegetarian, and vegan. BMI—body mass index.

**Figure A1:**
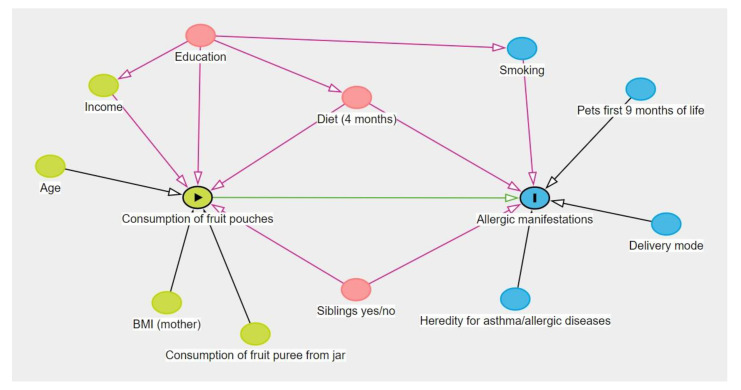
The image shows the directed acyclic graph constructed for finding potential confounders *a priori*. Shown in green are the variables possibly affecting the consumption of fruit pouches. The variables that possibly affect the development of allergic manifestations are shown in blue. Possible confounders, affecting both the consumption of fruit pouches and allergic manifestations are shown in pink.

## Data Availability

The data presented in this study are available from the corresponding author on reasonable request. Data are handled in accordance with the General Data Protection Regulation (GDPR) and are therefore not publicly available. The institutional review board and informed consent of the parents permit only the use of aggregated data to be published or made publicly available and prohibit sharing individual data.

## Appendix A

**Mother/partner start survey, gestational age 18–20 weeks. Response time: 4 weeks.**

Do you or your biological parents, siblings, or children have any of the following allergies (asthma, food allergy, allergy to furry pets, allergic rhinitis, eczema)? Yes/no.

What is the highest educational level that you have reached?

Response options:

Elementary school.

Lower/secondary school.

University.

**Mother survey gestational, age 26 weeks. Response time: 4 weeks.**

Have you smoked during this pregnancy?

Response options:

No.

Yes, I smoked in the last month before I got pregnant.

Yes, I smoked during the pregnancy.

**Mother/partner survey, gestational age 35 weeks. Response time: 4 weeks.**

What type of diet do you eat?

Response options:

I eat a mixed diet.

I eat meat but not fish.

I eat fish but not meat.

I eat meat but not pork.

I eat a vegetarian diet with milk and eggs (lacto-ovo-vegetarian).

I eat a vegetarian diet with milk but no eggs (lacto-vegetarian).

I eat a vegan diet.

**Child survey at 4 months of age. Response time: 3 weeks.**

Does the child share the home with siblings/other children? Yes/no.

Which type of milk is the child receiving now? Breastmilk, only formula, or both.

**Child survey at 9 months of age. Response time: 3 weeks.**

Does the child share the home with siblings/other children? Yes/no.

How often does the child meet with the following animals? (Cat, dog, rodents, birds, horse, farm animals.)

Response options:

Every day.

3–6 times/week.

1–2 times/week.

Rarely or never.

**NorthPop FFQ questions at 9 months of age** **.** **Response time: 3 weeks.**

During the past month, how often has your child eaten fruit or berry purée from a squeeze pouch?

Response options:

None.

1–3 times per month.

1–3 times per week.

4–6 times per week.

1–3 times per day.

4–6 times per day.

**NorthPop FFQ questions at 18 months of age. Response time: 3 weeks.**

Did the child ever wheeze? Yes/no.

Has the child received an asthma diagnosis from a physician? Yes/no.

Has the child ever had an adverse reaction to foods and drinks (e.g., rashes, vomiting, diarrhea, swelling of lips, runny nose or asthma)? Yes/no.

Has the child received a food allergy diagnosis from a physician? Yes/no.

Has the child had eczema? Yes/no. If yes, the following questions from the POEM score are asked:

Over the last week, on how many days has your child’s skin been itchy because of the eczema?

Over the last week, on how many nights has your child’s sleep been disturbed because of the eczema?

Over the last week, on how many days has your child’s skin been bleeding because of the eczema?

Over the last week, on how many days has your child’s skin been weeping or oozing clear fluid because of the eczema?

Over the last week, on how many days has your child’s skin been cracked because of the eczema?

Over the last week, on how many days has your child’s skin been flaking off because of the eczema?

Over the last week, on how many days has your child’s skin felt dry or rough because of the eczema?
